# HIV retesting in pregnant women in South Africa: Outcomes of a quality improvement project targeting health systems’ weaknesses

**DOI:** 10.4102/sajhivmed.v19i1.784

**Published:** 2018-07-12

**Authors:** Lauren M. Golden, Lee Fairlie, Freda Might, Stina Mojela, Dorothy Motsamai, Suzan Motshepe, Enoch Manyame, Craig Parker, Helen Rees, Gloria Maimela, Matthew F. Chersich

**Affiliations:** 1 Wits Reproductive Health and HIV Institute, Faculty of Health Sciences, University of the Witwatersrand, South Africa; 2 Aurum Institute, South Africa

## Abstract

**Introduction:**

South Africa is moving towards achieving elimination of mother-to-child transmission (eMTCT) but gaps remain in eMTCT programmes. Documenting successful outcomes of health systems interventions to address these gaps could encourage similar initiatives in the future.

**Methods:**

We describe the effectiveness of a Quality Improvement Project (QIP) to improve HIV retesting rates during pregnancy among women who had previously tested negative by redesigning the clinic process. Eight poorly-performing clinics were selected and compared with eight better-performing control clinics in a subdistrict in North West Province. Over nine months, root cause analysis and testing of change ideas using Plan-Do-Study-Act cycles were used to identify and refine interventions. Analysis of patient flow showed that women were referred for retesting following their nurse-driven antenatal visits, and many left without retesting as this would have further prolonged their visit. Processes were redesigned and standardised, where a counsellor was charged with retesting patients before antenatal consults. Staff were mentored on data collection and interpretation process. Quality improvement nurse advisors monitored indicators bi-weekly and adjusted interventions accordingly.

**Results:**

Retesting in intervention clinics rose from 36% in the three months pre-intervention to full coverage at month nine. At the end of the study, retesting in intervention clinics was 20% higher than in controls. Retesting also increased in the subdistrict overall.

**Conclusion:**

Service coverage and overall impact of HIV programmes can be raised through care-process analysis that optimises patient flow, supported by targeted QI interventions. These QI methodologies may be effective elsewhere for identifying new HIV infections in pregnant/breastfeeding women, and possibly in other services.

## Case report

In South Africa, great strides have been made towards the elimination of mother-to-child transmission (eMTCT) of HIV.^1,2^ Currently, a treat all approach is in place, including emphasising repeated HIV testing for women testing negative during pregnancy/breastfeeding, and lifelong antiretroviral treatment (ART) for HIV-positive women.^3^ Despite large investments in actualising these policies, some gaps in implementation remain.^2,4^ HIV retesting, in particular, needs strengthening given that incident HIV infection in women during pregnancy raises the risks of infant HIV transmission several fold.^5^

Interventions to strengthen health systems using quality improvement methodologies are key to raising programme performance and policy implementation^6^ and have been applied by PEPFAR partners in several districts.^7,8,9^ Documenting the effectiveness of these initiatives will further encourage their replication.

We describe the effectiveness of a Quality Improvement Project (QIP) to raise the performance of antenatal HIV retesting in the North West Province, where the HIV prevalence among pregnant women is around 29%.^2,10^

**FIGURE 1:**
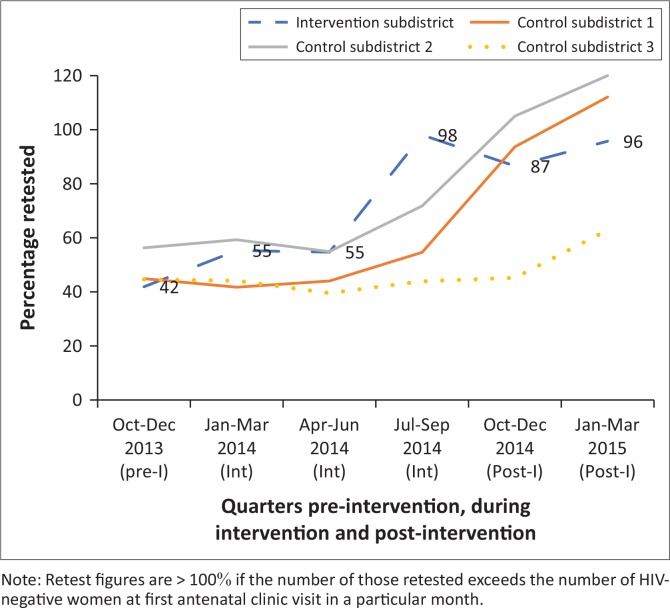
Antenatal clinics retesting rate in four subdistricts.

**FIGURE 2:**
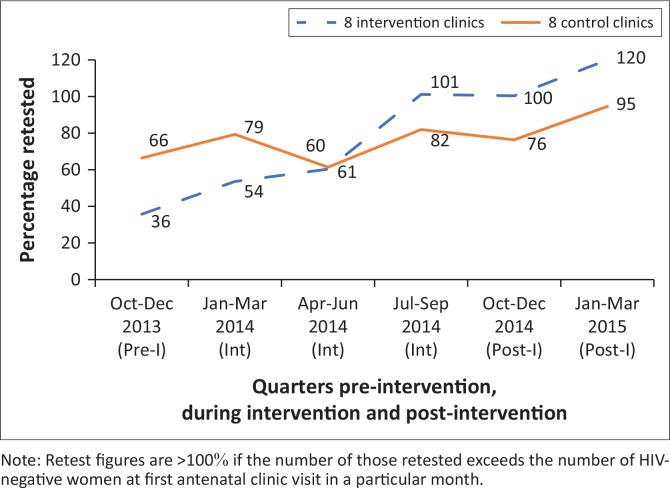
Antenatal clinics retest rate intervention versus control clinics.

The study compares eight intervention and eight control clinics in one subdistrict (Figure 1), set in an urban area surrounded by several large mines. To optimise the project’s impact, we purposively selected intervention sites, aiming to include those with low levels of retesting and high patient loads (selection based on data from the preceding three months). While selecting clinics with larger patient loads might optimise the interventions’ impact, it could bias comparisons between control and intervention sites as effectiveness of interventions may vary by clinic size. In the intervention clinics, the retest rate (number of HIV-negative women retested during pregnancy ÷ HIV-negative women at first antenatal visit) was 36% compared to 66% in control sites (Figure 2). The average number of women presenting to antenatal clinics (ANCs) was 3948 per month in intervention clinics and 3094 in controls. Also, the proportion of women attending ANC prior to 20 weeks gestation was 42% in intervention sites and 56% in controls.

A quality improvement team was established in each facility to conduct a QIP on retesting, which was implemented from January 2014 to September 2014. The ‘Model for Improvement’ was used to design the QIPs in intervention clinics.^11^ Root cause analysis was conducted using a fishbone tool and a process map, and Plan-Do-Study-Act (PDSA) cycles were used to test and adapt change ideas (interventions) over the 9-month period.^11^ Typically, a root cause analysis took 1–2 h, and often more than one session was required if there were many issues to ‘root out’. Quality improvement teams consisted predominately of facility staff (facility managers, nurses, counsellors, data capturers and clerks) and also local area managers, a data improvement advisor and a quality improvement nurse advisor (QIA). Quality improvement nurse advisors, who were mentors trained in implementation of quality improvement methodologies, were each assigned two of the intervention clinics. These advisors also worked in four of the control clinics, but on interventions other than retesting. The quality improvement team met weekly to review process outcomes, identify additional issues that needed to be ‘rooted out’ and rediscuss change ideas. Change ideas went through repeated testing cycles, which were monitored through a quality improvement tracker, which included process measures maintained by facility staff and QIAs including the use of diaries and allocation schedules.

The key finding of the root cause analysis was that, across all sites, women were being lost after referral to the counsellors for retesting, as this only took place following the women’s nurse-driven antenatal consultation. By then, women had already spent considerable time in the ANC and understandably did not wish to further prolong their visit. The main intervention, therefore, was to effect process redesign, whereby a counsellor was allocated to the ANC to identify and test eligible women as an integrated service within the clinic, and before women had their antenatal consultation. Women identified as HIV-positive in retesting commenced ART at that visit. Other interventions included in-service training on updated PMTCT guidelines, and mentorship provided by the data improvement advisor on recording, interpreting and reporting data. While the QIP methodology was standardised across intervention facilities, as was the process redesign, many other changes were specific to individual facilities, and evolved through repeated cycles of testing and adapting of changes until the desired results were achieved.

The antenatal clinic HIV retest rate indicators were monitored monthly using routinely collected District Health Information System (DHIS) data for each facility, as well as for the subdistrict as a whole. Run charts were plotted monthly for intervention and control clinics. Data were not available on whether women identified as HIV-infected on retesting began ART as this is not measured as a separate indicator on the DHIS (these women are subsumed in the total antenatal clients initiated on ART).

Post-intervention, the average ANC retest rate rose steadily from pre-intervention levels to about half that of control clinics to reach parity six months into the intervention. At month nine, retesting rates had reached full coverage in intervention clinics and were 20% higher than the controls, a difference that persisted for six months post-intervention (Figure 1). Moreover, many women who had not been retested in the period preceding the intervention now accessed these services (hence, figures exceeded 100%). Notable changes also took place in the retesting rates for the subdistrict as a whole, as well as in other subdistricts (Figure 2). At baseline, retesting rates were the lowest in the study subdistrict but had become the highest of the subdistricts at month nine. Although it is plausible that rises in rates in other subdistricts were related to the intervention, this cannot be ascertained with available data.

It is encouraging that improvements were sustained in the six months following the intervention, but long-term follow-up data are needed. Following the intervention, the QIP teams turned to providing support for PMTCT services more generally. As the facility staff had become proficient in QIP methodology, it is hoped that they would continue to not only apply QIP methods to retesting but also to other interventions that require strengthening.

In conclusion, the study indicates that process mapping, and simple, targeted QIP interventions were able to improve service coverage in the intervention clinics and the subdistrict as a whole. These findings are important as they again show the impact of health system interventions, especially those involving existing clinic staff, and address semi-urban settings, which are often understudied. Counsellor-based HIV testing prior to an antenatal consultation streamlined patient care, raised retesting rates and, by identifying women early after HIV infection, likely enabled timely initiation of ART for these women. This suggests that quality improvement approaches to health systems strengthening, such as that presented here, are central to efforts to raise the overall performance and impact of HIV programmes.

## Ethical consideration

The authors obtained ethical approval from the University of the Witwatersrand Human Research Ethics Committee (Ref R14/49, Protocol no. M151184) and the district health management team gave permission for study activities.
